# Quantitative CK19 biomarker detection in breast cancer cell lines

**DOI:** 10.25122/jml-2021-1101

**Published:** 2022-02

**Authors:** Zahra Orafa, Nasrin Karimi, Saeideh Keyvani, Mana Oloomi

**Affiliations:** 1. Molecular Biology Department, Pasteur Institute of Iran, Tehran, Iran

**Keywords:** breast cancer, molecular marker, CK19, cell lines, flow cytometry

## Abstract

Cytokeratin19 (CK19) was detected as the most related marker for circulating tumor cells, which was assessed in specific cell lines. MCF7, SKBR3, T47D, and MDA-MB-231, and HeLa cell line as negative control were used. CK19 expression was confirmed by using mouse monoclonal anti-human CK19 antibody. CK19 detection in MDA-MB-231 was not observed. CK19 marker expression was compared in T47D, MCF7, and SKBR3 cell lines. T47D and MCF7 belonged to the luminal subtype of breast cancer (BC) that CK19 expression regulated with an ER marker. SKBR3 belonged to the HER2 positive subtype of BC. However, MDA-MB-231 belonged to the claudin-low subtype of BC that lack of CK19 expression strongly is related to negative ER, PR, and HER2. Therefore, there are not only quantitative differences in CK19 expression, but its expression could also link to the other markers of BC that should be considered in the molecular classification of breast carcinoma. Different expression levels related to cell classification could be useful in the prognosis and treatment of cancers with epithelial origins.

## Introduction

Breast cancer is one of the most common cancers in women around the world [1]. Malignant growth of the epithelial cells covering the ducts or glands of the breast tissue is the main cause of this cancer [2]. Despite many advances in early detection and treatment, this disease is still one of the most important death causes among women [3]. Analysis of gene expression has classified five different subtypes in breast cancer: Luminal A, Luminal B, HER2 (Human epidermal growth factor receptor-2) positive, Basal-like, and Normal-like/Claudin-low [4–6]. Each subtype illustrates a different prognosis, metastasis patterns, and different responses to treatment [7]. Luminal A subtypes of breast cancer are positive in the expression of estrogen (ER) and progesterone (PR) receptors and negative in the expression of HER2 (ER^+^/PR^+^/HER2^–^) and also shows the expression of Cytokeratin (CK) markers such as CK7, CK8, CK18 and CK19 [4, 5]. Liminal B subtypes of breast cancer are positive in the expression of ER, PR, and HER2 receptors (ER^+^/PR^+^/HER2^+^) and the expression of markers like CK7, CK8, CK18, and CK19 and show an up-regulation of genes related to cell proliferation [4, 5]. HER2 positive subtypes of breast cancer are negative in terms of ER and PR receptors expression and are positive for HER2 expression (ER^–^/PR^–^/HER2^+^) and express markers such as CK5, CK8, CK18, and CK19 [7–9]. The basal-like subtypes of breast cancer are negative for the expression of ER, PR, and HER2 (ER^–^/PR^–^/HER2^–^) and express CK5, CK6, CK14, CK17 [5, 9, 10]. This subtype is called triple-negative because they do not show expression of PR, ER, and HER2 [11]. The Normal-like subtype of breast cancer is negative for ER, PR, and HER2 (ER^–^/PR^–^/HER2^–^), and there is no expression for CK8, CK18, and CK19 markers [7, 9]. The patients of this subtype are different from other subtypes; they are responsive to treatment [12]. The phenotype of this subtype is similar to the non-cancerous breast tissues, but its features have not been clearly specified [5, 13]. The Claudin-low subtype of breast cancer is negative in the expression for ER, PR, and HER2) ER^–^/PR^–^/HER2^–^) and typically expresses the CK5, CK14, and CK17 markers [7, 14]. Due to the lack of expression of PR, ER, and HER2, this subtype is also called the triple-negative [12].

CKs are the major structural proteins in epithelial cells [15]. They are a member of the family of intermediate filaments. CKs normally cause the organization of the cytoskeleton, but its abnormal expression can push the cells to become cancerous [16]. CK19 is the smallest member of the CK family [17]. This epithelial marker was identified from squamous cell carcinoma for the first time [18]. Despite other CKs that create heterodimer structures, CK19 does not create a heterodimer structure with any CKs; therefore, it is called simple CK [19, 20]. CK19 also has high expression in metastatic cancers, including breast, liver, lung, pancreas, and esophageal [21]. It has different functions in maintaining the structure of the cell [22, 23], in cellular communications [24], apoptosis [25], regulating the synthesis and transferring protein [17].

Furthermore, epithelial cells from initial tumors can be identified in the peripheral blood of breast cancer patients, which these cells are known as circulating tumor cells (CTCs) [26]. CTCs are the first detectable cells with metastatic capabilities as a biomarker for breast cancer prognosis. It is mentioned that CKs are the best identification markers for CTC [27]. CK19 is recognized as a sensitive marker to detect early metastasis and cancer prognosis in tumor cells with epithelial origin in the blood [28]. The data supported the possibility that CK19 detection in patients’ blood could be a marker for breast cancer detection [10].

Additionally, breast cancer cell lines are the reproducible sources widely used to discover biological and clinical functions such as studying tumors, the signal transduction pathways, and modern therapeutic goals [7]. They were primarily used as the breast cancer experimental models in many types of cancer research [8]. The first human cell line was established from cervical cancer cells (HeLa cells), then human breast cancer cell lines from pleural effusion metastases were used [9]. The MCF7 cell line was obtained from the metastatic cells of pleural effusions [10]. MCF7 is the most widely studied breast carcinoma cell line because of its steroid receptor status and ER sensitivity [11]. MCF7 cells are tumorigenic in nude mice, and it is ER^+^, PR^+^, and CK19 positive [12]. The T47D cell line was also isolated from the pleural effusion of a woman diagnosed with ductal carcinoma [13]. T47D cells are not tumorigenic; they are ER^+^, PR^+^, and express E-cadherin [14]. This cell line is widely used as the experimental model for breast cancer studies [15]. The SKBR3 cell line has a prominent feature of the HER2 high expression cell line [16]. The MDA-MB-231 cell line was from a woman with ductal carcinoma, and it was negative for the expression of PR, ER, and HER2 genes with tumorigenicity in nude mice [17].

There is a necessity for finding a common biomarker that could be used in experimental and clinical research. In this study, CK19 marker expression was accessed in characterized cell lines as the most related marker for circulating tumor cells (CTCs), and its relation with other defined markers was considered for the first time. The research has been performed to quantify CK19 expression in different cell lines by flow cytometry used in the research literature. CK19 detection assessment in patients’ blood and cell lines research could support and explain data variation in breast cancer therapy results, which can improve future treatment procedures.

## Material and Methods

### Cell Culture

Human breast cancer cell lines (MCF7, SKBR3, MDA-MB-231, and T47D) and cervical cancer cell line (HeLa) as negative control were obtained from the cell bank of Pasteur Institute of Iran. Briefly, cell lines were cultured in RPMI 1640 (Gibco) supplemented with 10% Fetal bovine serum (FBS, Invitrogen) and penicillin-streptomycin (Biosera). First, the cells were kept at 37°C in a humidified CO_2_ incubator (5% CO_2_). Then, cell lines grown in monolayer were harvested by washing once with phosphate-buffered saline (PBS), pH 7.3, and then incubating the cells with trypsin/EDTA (Biosera) for 2–5 min at 37°C. Finally, cells were counted using a hemocytometer.

### Western blot analysis

Cell lysis was performed by resuspending 0.5 × 10^6^ cells from each cell lines in 50 μl PBS (Phosphate buffer saline) 1X at 4°C. The protein concentration of the samples was determined using Picodrop. Then, Lysates of cell lines were mixed with Laemmli sample buffer [18] and incubated at 95°C for 20 min. Equal amounts of protein (10 μg) were separated by sodium dodecyl sulfate-polyacrylamide gel electrophoresis (SDS-PAGE) on 12% polyacrylamide gel and then transferred to nitrocellulose membrane (Whatman). The membrane was stained with Ponceau S (Merck) to evaluate transfer efficiency. Then protein binding sites on the nitrocellulose were blocked with skim milk in 1% PBS-Tween 20 (PBST) at 4°C overnight. The membrane was washed three times with PBST and then incubated for 2 hours at room temperature with mouse monoclonal anti-human CK19 antibody (ABD Serotec, diluted 1:1000 in PBS 1X) as a primary antibody. Mouse monoclonal anti-β-actin (Sigma, diluted 1:1000 in PBS 1X) was also used as an internal control. After washing three times with PBST, the membrane was incubated with anti-mouse IgG- peroxidase-conjugated (Sigma, diluted 1:4000 in PBS 1X) as a secondary antibody at room temperature for 1 h. The membrane was washed three times with PBST, and the protein bands were detected by Enhanced Chemiluminescence (ECL, Amersham) kit then exposed to X-ray film.

### Flow cytometric analysis

The expression of CK19 in breast cancer cell lines was determined by flow cytometry. In this regard, 1 × 10^6^ cells from each cell line were fixed with 4% paraformaldehyde (Merck) in PBS 1X for 20 min at room temperature. Then, cells were washed twice with 1% PBS with FBS and were permeabilized with ice-cold 100% methanol (Merck) for 30 min at 4°C. Cells were washed twice with 1% PBS-FBS and blocked with 1% bovine serum albumin (BSA) for 45 minutes at room temperature. Cells were washed twice with 1% PBS-FBS and then were stained with Fluorescein isothiocyanate (FITC)-conjugated mouse anti-human CK19 antibody (Abcam, diluted 1:300 in PBS 1X) or FITC-mouse IgG2a isotype antibody (Abcam, diluted 1:300 in PBS 1X) for 1h at room temperature as a negative control. Finally, they were washed twice with 1% PBS- FBS, and detection of bound antibodies was determined by flow cytometry (Cyflow). The results were analyzed with the FlowJo® program.

### Immunofluorescence microscopy

CK19 expression in the MCF7 and T47D cell lines was also detected by immunofluorescence assay. Cultured cells were assessed on glass slides. They were fixed with 4% paraformaldehyde (Merck) in PBS 1X for 20 min at room temperature. The cells were washed twice with 1% PBS-FBS and were permeabilized with ice-cold 100% methanol (Merck) for 30 min at 4°C. Cells were washed twice with 1% PBS-FBS and blocked with 1% bovine serum albumin (BSA) for 45 min at room temperature. Cells were washed twice with 1% PBS-FBS, and then Immunostaining was done with the FITC-conjugated mouse anti-human CK19 antibody (Abcam, diluted 1:300 in PBS 1X). Then FITC-mouse IgG2a isotype antibody (Abcam, diluted 1:300 in PBS 1X) as a negative control was performed for 1h at room temperature. The cells were washed twice with 1% PBS-FBS, and detection of CK19 protein was determined under a fluorescent microscope (Leitz Laborlux).

## Results

### Cell lines culture (MCF7, SKBR3, MDA-MB-231, T47D, and HeLa) 

MCF7, SKBR3, MDA-MB-231, T47D, and HeLa cell lines were purchased from the cell bank of Pasteur Institute of Iran, and after defrosting, they were cultured in the medium. In order to obtain the same amount of cells for western blot and flow cytometry analysis, the cells were cultured several times, and the count was carried out by Neubauer slide.

### CK19 expression in breast cancer cell lines by western blot

**Figure 1.**
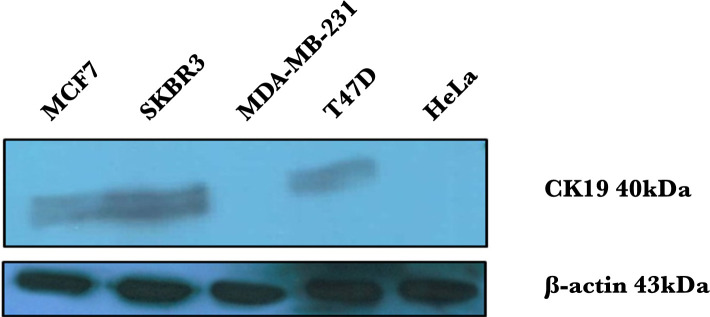
CK19 expression was shown by western blot analyses in breast cancer cell lines. 20 μg of each cell line protein was applied in the 12% Acrylamide gel. After transferring to the nitrocellulose membrane, the membrane was incubated using mouse monoclonal CK19 antibody and HRP (horseradish peroxidase) anti-mouse antibody. Then, the protein bands were revealed by the ECL method. The cervical cancer cell line (HeLa) was considered as a negative control.

To detect the expression of the CK19 protein in breast cancer cell lines, western blot analysis was performed. In this study, the cervical cancer cell line (HeLa) was also used as a negative control. First, the expression of the β-Actin protein was investigated as a normalizing factor. The nitrocellulose membrane was incubated with the mouse monoclonal β-Actin antibody (primary antibody) and then with the HRP anti-mouse antibody (secondary antibody). Finally, the protein bands were revealed by the ECL technique. β-Actin protein detection showed the same expressions in the cell lines. In order to investigate CK19 expression, we used mouse monoclonal CK19 antibody as the primary antibody and the HRP anti-mouse antibody as the secondary antibody. The CK19 antibody was specific for this marker, and it was from the lgG1 class and BA17 clone. The results obtained from western blot indicated the different levels of CK19 expression in breast cancer cell lines. The SKBR3, MCF7, and T47D cell lines were all positive in terms of expression of CK19, while no expression of this protein was shown in the MDA-MB-231 cell line (Figure 1). In fact, the cell lines from Luminal subtypes of breast cancer (MCF7 and T47D) and subtypes of HER2 positive of breast cancer (SKBR3) indicated CK19 expression, while there was no expression in the cell line of the Normal-like/claudin-low subtypes of breast cancer (MDA-MB-231).

CK19 expression in cell lines from the Luminal subtypes of breast cancer (MCF7 and T47D) and the HER2 positive subtypes of breast cancer (SKBR3) was detected, while no expression of this protein was detected in cell lines from the other subtypes same as Normal-like/claudin-low (MDA-MB-231). Furthermore, the cell lines from Luminal subtypes of breast cancer had a higher expression of CK19 compared to the cell line of HER2 positive subtypes of breast cancer.

### CK19 expression in breast cancer cell lines by flow cytometry

**Figure 2.**
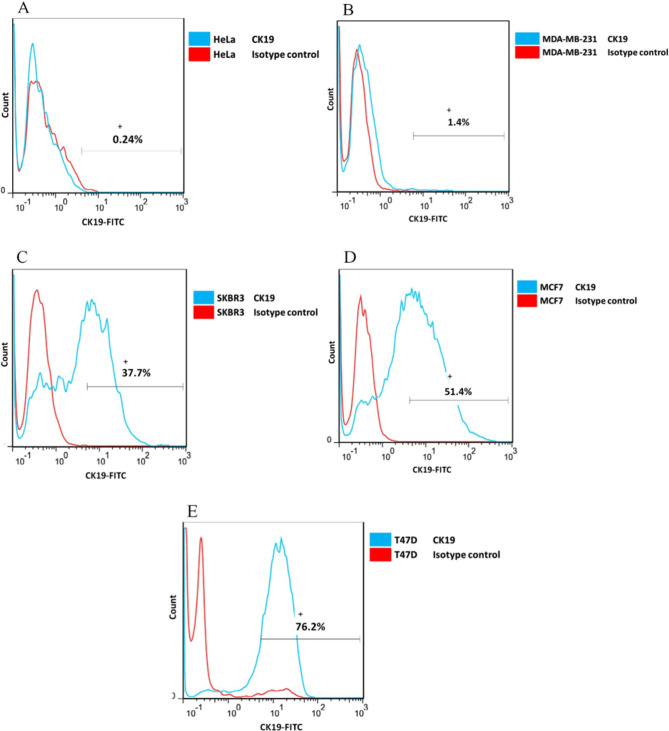
Breast cancer cell lines were fixed with 4% paraformaldehyde, then permeabilized with 100% methanol. After being blocked with the blocking buffer, they were colored with the mouse monoclonal antibody CK19 conjugated to FITC and the mouse monoclonal antibody lgG2a conjugated to FITC (isotype control). The cervical cell line (HeLa) was considered as a negative control. Cell lines: (A) HeLa; (B) MDA-MB-231; (C) SKBR3; (D) MCF7; (E) T47D. CK19 expression in the MDA-MB-231 cell line was not detected, while 37.7, 51.4, and 76.2% mean fluorescence of expression was detected in SKBR3, MCF7, and T47D cell lines, respectively.

The expression of the CK19 marker in breast cancer cell lines was investigated by flow cytometry. In this experiment, the cervical cancer cell line (HeLa) was considered as the negative control. 1×10^6^ cells of each cell line were colored with the mouse monoclonal CK19 antibody, linked to FITC, and 1×10^6^ cells of each cell line were colored with the mouse monoclonal IgG2a antibody, linked to FITC (isotype control). The CK19 antibody is specified for this marker, and it is from IgG2a class and A53-B/A2 clone. The obtained results from flow cytometry analysis indicated the different levels of CK19 marker expression in the studied cell lines. CK19 expression in the MDA-MB-231 cell line was not detected, while 37.7, 51.4, and 76.2% expression were detected in SKBR3, MCF7, and T47D cell lines, respectively. In the SKBR3 cell line, the least expression, and in the T47D cell line, the most expression level was detected. CK19 marker expression for the negative control HeLa cell line was not detected by flow cytometry (Figure 2).

### CK19 expression in MCF7 and T47D cell lines by immunofluorescence microscopy

**Figure 3.**
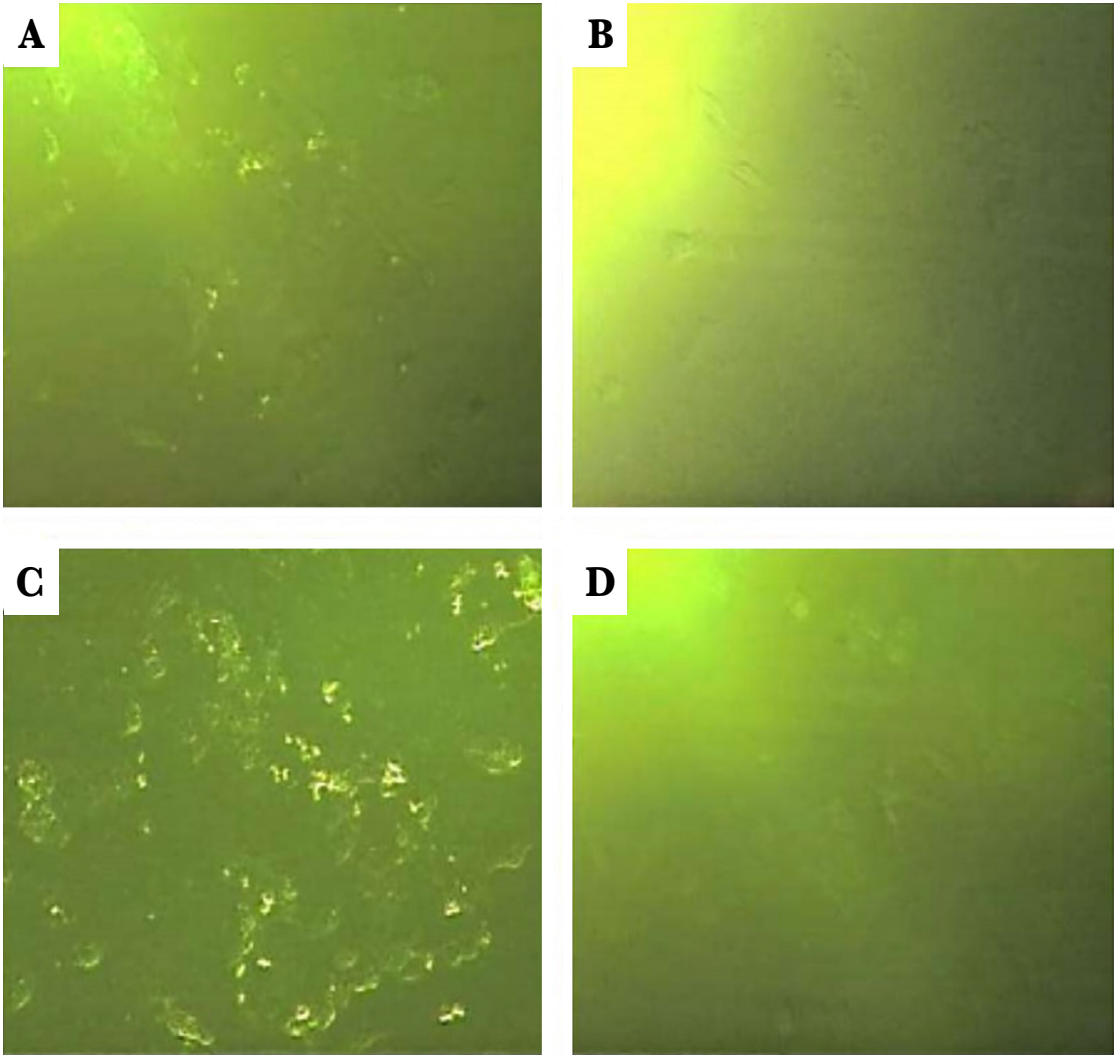
CK19 expression in the MCF7 and T47D cell lines was demonstrated by immunofluorescence microscopy. The cell lines were fixed with 4% paraformaldehyde and then were permeabilized by 100%methanol and blocked with the blocking buffer. They were colored by the mouse monoclonal CK19 antibody conjugated with FITC and the mouse monoclonal lgG2a antibody linked to conjugated FITC (isotype control). Cell lines with 10X magnifications: A – MCF7 with CK19 antibody; B – MCF7 with isotype control; C – T47D with CK19 antibody; D – T47D with isotype control.

The microscopic immunofluorescence detection was done to confirm the CK19 expression in MCF7 and T47D cell lines. For each cell line, two cell culture plates were considered. One of the plates was incubated with CK19 monoclonal antibody conjugated to FITC, and the other one was incubated with mouse lgG2a antibody (as the isotype control). The cell lines were investigated by the wavelength of 488 nm with the immunofluorescence microscope (Figure 3).

## Discussion

Breast cancer is a very heterogeneous disease at molecular, histopathological, and clinical levels. In addition, established cell lines derived from the primary or metastatic cancers still provide an important experimental tool for biological cancer studies and genetic modifications *in vitro* and *in vivo* [19]. The heterogeneity in the molecular level has been indicated in gene expression among breast cancers. The studies based on the whole genome analysis using the microarray method led to discovering five distinct subtypes of breast cancer, Luminal A, Luminal B, Her-2 positive, Basal-like, and Normal-like/Claudin-low. The subsequent studies based on the immunohistochemical method and by using antibodies against ER, PR, HER2, and CK markers revealed the properties of these subtypes. These subtypes respond differently to treatments and also have different metastatic patterns [3]. At present, more than 20 different CKs have been identified out of which CK19 is one of the most significant markers in simple epithelial cells, breast, prostate, lung, colon, and ovary cancers [20].

In this study, flow cytometry was used to quantify CK19 expression in T47D, MCF7, SKBR3, and MDA-MB-231 cell lines. The antibody against CK19 was provided from the A53-B/A2 clone for flow cytometry and the BA-17 clone for western blot. It was indicated that the CK19 marker has no expression in the HeLa cell line; therefore, this cell line was used as the negative control in this study [21]. CK19 marker expression in T47D and MCF7 cell lines was quantitatively indicated in this study. Riaz *et al.* studied T47D and MCF7 cell lines by microarray technique and indicated that both cell lines show ER and PR receptors expression and are negative in the expression of HER2 (ER^+^/PR^+^/HER2^–^); hence, they are considered Luminal subtypes [3]. ER and its ligand interact with the DNA sequence region called the Estrogen response element (ERE). CK19 marker expression in two T47D and MCF7 cell lines was detected and showed that their origin is from luminal epithelial cells [22]. The internal breast cell layer includes the epithelial (luminal) cells, and the external layer includes myoepithelial (basal) cells. The cells of the luminal layer express markers such as ER, CK8, CK18, CK19, and MUC1 [23]. In the SKBR3 cell line in this study, the expression of the CK19 marker was also indicated quantitatively. The microarray technique showed that this cell line is positive for HER2 expression and negative for ER and PR receptors (ER^–^/PR^–^/Her-2^+^) [24–27]. Therefore, the expression regulation of the CK19 marker in this cell line is under the influence of the HER2 signaling pathway. HER2 levels in human breast tumor tissue were also strongly supposed to correlate with CK19 expression levels. The expression of CK19 is modulated by HER2 signaling at the level of transcription. The CK19 expression is also upregulated in HER2 overexpressing cells. CK19 was upregulated at the transcriptional and translational level in high-HER2 expressing breast cancer cells, suggesting that HER2 expression is coupled with CK19 expression. Using western blot analysis, Zhang *et al.* indicated the CK19 expression in HER2^+^ tumors and concluded that HER2 influences tumor cells with invasion and metastasis by regulating CK of cytoskeleton’s expression [28]. Using flow cytometry and western blot methods, Alix-Panabières *et al.* also indicated that the expression of the CK19 marker in MCF7 and SKBR3 cell lines gives a high metastatic feature to these active cells [23]. Bock *et al.*, with a microscopic investigation of SKBR3, MCF7, and T47D cell lines, indicated that all three cell lines express CK19; but the MCF7 and T47D cell lines show the expression of ER while SKBR3 cell line shows the expression of HER2 [29]. We showed the expression of the CK19 marker in three cell lines of SKBR3, MCF7, and T47D with the western blot method in the same condition. By flow cytometry, this study showed that the expression of this marker in MCF7 and T47D cell lines is more than SKBR3. Fujisue *et al.* stated that compared to HER2, the complex of ER hormone-receptor has a stronger impact on the expression of the CK19 marker [30]. CK19 marker expression in the MDA-MB-231 cell line was not detected in this study. Riaz *et al.* studied this cell line with microarray technique and indicated that it is negative in terms of the expression of ER, PR, and HER2 receptors (ER^–^/PR^–^/HER2^–^); thus, it is located in the Normal-like/Claudin-low subtype [3]. The lack of expression of this marker in this cell line is significantly related to the lack of expression of PR, ER, and HER2 [28]. On the other hand, the Epithelial-Mesenchymal Transition (EMT) process in this cell line is another issue that could lead to the lack of CK19 expression [29]. MDA-MB-231 cell line, derived from human breast carcinomas that do not express ER, is often used as an experimental non-hormone dependent tumor model [30]. EMT is the process in which the cancer cells lose their epithelial properties and adopt mesenchymal features. During this process, the cytoskeleton is reorganized, and the cell loses its epithelial markers such as CK19 and E-cadherin and qualifies expression of mesenchymal markers like Vimentin. EMT leads to the invasiveness and migration of the tumor cells toward the target tissue, which is the reason for tumor advancement and metastasis [31]. Expression of CK19 has been lost in Normal-like subtypes of breast cancer under the EMT process [32]. The process of becoming invasive and metastatic is the result of CK19 reduction of expression [25]. For the same reason, the MDA-MB-231 cell line is considered a metastasis model, known as the triple-negative [31]. By using the western blot method, Mladkova *et al.* found that the CK19 marker is expressed in the MCF7 cell line but not in the MDA-MB-231 cell line, and they stated that while both cells have the same origin; their phenotype is different [32]. Up to now, three monoclonal CK19 antibodies named BA17, BA16, and A53-B/A2 have been used to detect the expression of this marker. For the first time, tissues were investigated with the immunohistochemical method, and different expression of CK19 was reported by the pathological epithelial studies [33].

Different techniques and markers are currently used to identify the cancer cells in blood samples with monoclonal antibodies. However, flow cytometry-based studies provide valuable information for the diagnosis, treatment, and prognosis of breast cancer patients, especially for identifying the tumor cells existing in the blood [34]. Wang *et al.* illustrated that CK19 detected in the peripheral blood by flow cytometry is typically an easy and certain method to find the CTCs in patients with breast cancer, the primary metastasis, or disease progress [12]. In 2011, CK19 marker expression was detected among Iranian women with breast cancer by the RT-PCR method [35]. CK19 expression was significantly reported differently among patients and healthy people and even in different stages of the disease [36, 37].

The CK19 metastatic potential has been an interest in recent years. Its application as a tumor marker among 30 cancer cell lines such as thyroid, thoracic, lung, pancreatic, cervical, colorectal is important to consider [38].

## Conclusions

This is a more comprehensive report of different levels of CK19 expression based on molecular cell lines classification that helps in choosing the right cell line in breast cancer research. In this study, research has been performed to add the quantification of CK19 expression in different cell lines by flow cytometry used in the research literature. Blood specimens were tested by flow cytometry to quantify the expression of CK19. Different expressions of CK19 were studied in human breast cancer cell lines, SKBR3, BT549, and BT474, which are used in most literature articles regarding breast cancer.

Another detection technique, western blotting, was also used in this study for confirming the protein expression. CK19 expression in the MDA-MB-231 cell line was not detected, while 37.7, 51.4, and 76.2% mean fluorescence expression was detected in SKBR3, MCF7, and T47D cell lines, respectively. In conclusion, flow cytometry is an easy and reliable method for measuring the level of CK19 marker expression that could indicate the expression regulation of other markers in breast cancer cells. Therefore, it can be concluded that CK19 expression and its regulation by ER hormone or HER2 signaling pathway could be useful in the prognosis and treatment of cancers with epithelial origins.

## Acknowledgments

### Conflict of interest

The authors declare that there is no conflict of interest.

### Ethics approval

The approval for this study was obtained from the National Ethical Committee of the Pasteur Institute of Iran (Ethical approval No. 4552). This article is based on a thesis submitted to the graduate office in partial fulfillment of the requirements for the degree of MSc student.

### Consent to participate

The informed consent written form was obtained from each participant.

### Funding

Pasteur Institute of Iran funded the thesis.
